# Current challenges and emerging opportunities of chimeric antigen receptor-engineered cell immunotherapy

**DOI:** 10.1186/s40164-025-00683-y

**Published:** 2025-07-02

**Authors:** Yong Liu, Yifei Duan, Zefan Du, Bo Lu, Su Liu, Lindi Li, Mengyao Tian, Liang Li, Ran Yao, Cheng Ouyang, Mo Yang, Chun Chen

**Affiliations:** 1 Pediatric Hematology Laboratory, Division of Hematology/Oncology, Department of Pediatrics, The Seventh Affiliated Hospital of Sun Yat-Sen University, Shenzhen, 518107 Guangdong China; 2 Scientific Research Center, The Seventh Affiliated Hospital of Sun Yat-Sen University, Shenzhen, 518107 Guangdong China; 3 Department of Haematology, The Seventh Affiliated Hospital of Sun Yat-Sen University, Shenzhen, 518107 Guangdong China; 4 Department of Hematology, Affiliated Hospital of Guangdong Medical University (GDMU), Zhanjiang, 524001 Guangdong China

**Keywords:** CAR-T, CAR-NK, CAR-M, Immunotherapy, Clinical application

## Abstract

Chimeric antigen receptor (CAR) engineered cellular immunotherapy offers the potential for precise targeting and elimination of tumor cells, providing a tailored approach to cancer treatment. CAR-T cells demonstrate significant anti-tumor activity among these therapies. Nonetheless, these therapies may trigger adverse effects, including inflammatory and neurotoxic reactions during treatment. Recent efforts have been directed toward enhancing efficacy by optimizing CAR design or modulating its activity. Compared to CAR-T cells, CAR-engineered natural killer cells (CAR-NK) present notable advantages, including various sources and diminished toxicity, and are gaining recognition in clinical research. CAR-macrophages (CAR-M), while sharing antigenic domains similar to those of CAR-T cells, display superior capabilities in antigen presentation and tumor penetration. As a result, there is significant enthusiasm surrounding investigations into CAR-NK and CAR-M cell immunotherapies. This review explores the existing environment and obstacles associated with immunotherapies that utilize CAR-T, CAR-NK, and CAR-M cells to inspire novel pathways for forthcoming clinical applications.

**Supplementary Information:**

The online version contains supplementary material available at 10.1186/s40164-025-00683-y.

## Introduction

Cancer continues to pose a significant threat to global health, with both its incidence and mortality escalating each year. Projections by the World Health Organization’s (WHO) International Agency for Research on Cancer suggest that by 2050, cancer incidence will surge by 77% from 2022 levels, with the number of cases approaching 35 million, underscoring the critical need for targeted therapeutic approaches [1]. While traditional modalities such as surgery, chemotherapy, and radiotherapy are prevalent, they frequently fail to markedly improve life quality for patients with metastatic or recurrent forms of cancer [2, 3]. Compared with traditional chemotherapy drugs, small molecule targeted drugs have significant advantages in efficacy, but they still have problems with low efficiency and drug resistance [4]. In this context, cancer immunotherapy has arisen as a pivotal component of contemporary cancer treatment strategies [5]. This field encompasses strategies such as therapeutic cancer vaccines [6], immune checkpoint inhibitors (ICIs), oncolytic virus (OV), proteolysis targeting chimera (PROTAC) [7], and adoptive cellular immunotherapy (ACI), all designed to catalyze T cell-mediated responses and bolster the synergy between innate immune components, especially antigen-presenting cells, and immune effectors [8, 9].

Currently, the predominant immunotherapies in clinical use include ICIs and chimeric antigen receptor T cells (CAR-T) therapy, the latter representing a specialized type of ACI. Over 1,700 clinical trials involving CAR-T therapy have been cataloged on the Global Clinical Trials Registry Platform. Although initially concentrated on hematological malignancies, extensive research is now also targeting the application of CAR-T cell therapy in treating solid tumors. The U.S. Food and Drug Administration (FDA) has approved several CAR-T cell therapies, specifically for treating recurrent or resistant B-cell malignancies [7, 10]. As clinical trials of CAR-T cells progress, there is increasing examination of significant adverse effects such as central neurotoxicity, cytokine release syndrome (CRS), hematopoietic suppression, and infections. Furthermore, the efficacy of CAR-T therapies in targeting solid tumors continues to present significant challenges [11]. Consequently, scientists are exploring alternative immunotherapeutic approaches like CAR-engineered natural killer cells (CAR-NK) and CAR-macrophages (CAR-M), which employ diverse cell types such as autologous, allogeneic, xenogeneic, and transgenic cells to trigger distinct anti-cancer immune responses. However, alternative treatments such as CAR-NK and CAR-M remain largely within the realm of preclinical investigation [12]. Moreover, there is presently no fully developed CAR-engineered cell therapy for patients with solid tumors, indicating significant room for advancement in this area of medical research. This paper seeks to thoroughly analyze developments in CAR-engineered cell therapies, including CAR-T, CAR-NK, and CAR-M cells, to enhance understanding of their potential in advancing oncological treatment methods in immunotherapy.

## CAR structure

T cell activation necessitates two critical signals. The first is an antigen-specific signal, which is triggered when the T cell receptor (TCR) recognizes a specific peptide presented by the major histocompatibility complex (MHC) on antigen-presenting cells. The second is a co-stimulatory signal, typically mediated by the interaction between CD28 on T cells and its ligands, B7.1 (CD80) or B7.2 (CD86), on antigen-presenting cells [13, 14]. Antigen recognition and binding are facilitated by the two highly variable chains that make up the TCR. One fraction, γδT cells, consists of a γ chain and a δ chain, while the majority of mature T cells, called αβT cells, are made up of an α and a β chain [15, 16]. CAR holds specific advantages over TCR. Unlike the αβT cell, swhich requires MHC-dependent recognition of antigens, CAR can operate independently of MHC, targeting both protein and non-protein molecules expressed on the cell surface to activate T cell effector functions [17]. More importantly, traditional T cell therapy is often limited in effect due to exhaustion caused by the tumor microenvironment and poor antigen recognition, while CAR engineering enables CD4 + and CD8 + T cell subsets to work synergistically to completely eliminate tumors. Studies have shown that CD4 + CAR-T cells can activate antigen-presenting cells to enhance endogenous anti-tumor immunity [18], while CD8 + CAR-T cells maintain long-term memory responses [19]. This functional complementarity can improve the persistence of the two subsets, highlighting the unique therapeutic potential of CAR-T technology [20].

**Fig. 1:**
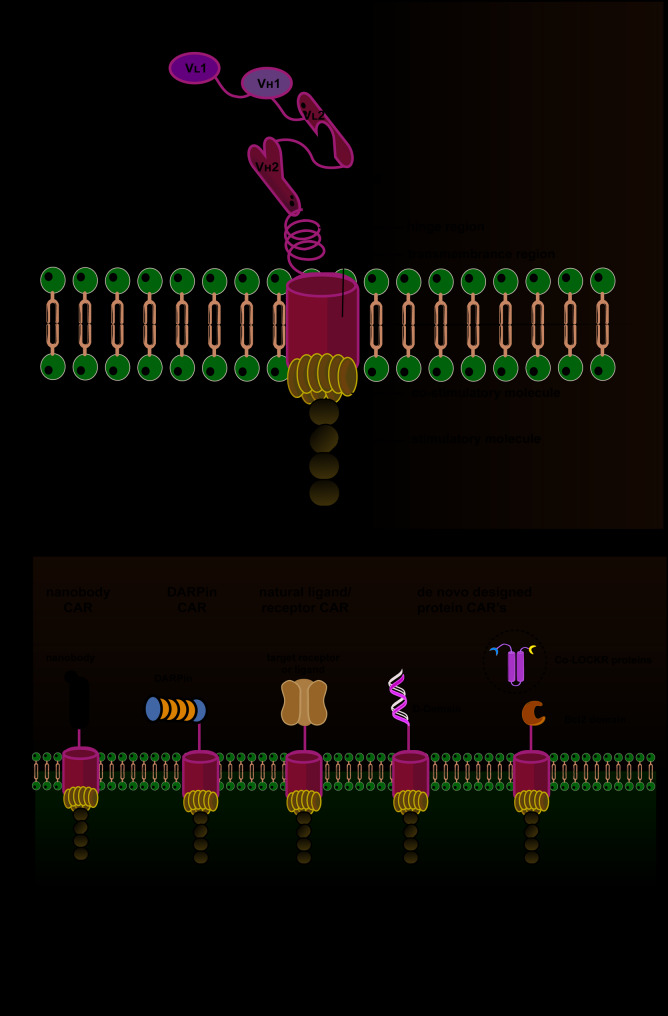
The Structure of CAR. **A**) A CAR is engineered with four fundamental components: extracellular antigen-binding domains, hinge regions, transmembrane domains, and intracellular signaling domains. **B**) Various Non-canonical CARs that use non-scFv binding domains are depicted, including nanobodies, DARPins, natural ligands or receptors, and de novo-designed synthetic proteins including D-domains and Co-LOCKR

A CAR is engineered with four fundamental components: extracellular antigen-binding domains, hinge regions, transmembrane domains, and intracellular signaling domains (Fig. 1A) [21].Currently, most CAR structures use a single-chain variable fragment (scFv) to achieve antigen recognition, but it is not the only option for CAR antigen targeting. Researchers have recently turned their attention to atypical protein binding domains (Fig. 1B). Unlike the common light and heavy chain pairing in traditional antibodies, nanobodies (also known as variable domains of the heavy chain of heavy-chain antibodies, VHH) have the unique ability to fold into monomers. It is only about 110 amino acids in size and is a compact IgG-like protein binding domain [22]. Hasannia’s team used anti-transmembrane glycoprotein mucin 1 (MUC1) nanobody as the antigen binding domain, CD28 and CD3ζ as the signaling domain, and IgG3 as the spacer in the chimeric receptor construct to construct a nanobody chimeric receptor, and confirmed that it has the function of targeting tumor-associated antigen-positive cells [23]. The team also further confirmed in subsequent studies that VHHs can be used as a good substitute for scFv [24]. In addition, Hammill et al. used the designed ankyrin repeat protein (DARPin) as a tumor antigen targeting domain and confirmed that DARPin CARs can trigger cytotoxic responses in both mouse and human systems, and the effect is similar to that of scFv CARs. This discovery has opened up a new way to construct CARs with new antigen binding properties [25]. Natural receptor-based CARs, including designed ankyrin repeat protein (DARPin), NKp30, DNAM-1, CD27, CD16, and ligand-based CARs, including GM-CSF, Adnectin, FSH, and T1E, are being developed continuously [26, 27]. De novo–designed synthetic protein elements, including the D-Domain binder and the Co-LOCKR logic-gating protein system, are also used to construct CAR structures [28, 29]. Research on available CAR domains is still ongoing.

## CAR-T cell therapy

### Evolution of CAR-T cells

CARs were initially integrated into T cells to boost their capacity for precise target cell identification and potent cytotoxic effects [30]. Employing T cells modified for TCR-independent activation, CAR-T cell immunotherapy represents a type of adoptive cell transfer [31].As early as the 1980s, Kuwana et al. first described CAR’s structure by using a chimeric gene composed of an immunoglobulin-derived variable (V) region and a TCR-derived constant (C) region.The results showed that these altered T cells could target and kill trinitrophenyl (TNP)-coated cells without relying on MHC [32]. The first generation of CAR-T cells was made possible by this revolutionary study [33]. There has been significant advancement in cancer treatment with the approval of seven CAR-T cell treatments worldwide so far.

#### First-generation CAR-T cells

When it comes to CAR-T cell treatment, CD19, CD22, and B cell maturation antigen (BCMA) are the main targets. In most cases, CAR-T cell generation takes around 10 to 14 days, and the dosages given range from 1 × 10^4^ to 1 × 10^10^ cells/kg [34]. To imitate normal cell signaling, first-generation CAR-T cells attempt to combine an external antigen recognition domain with an intracellular CD3ζ signaling domain [32]. However, first-generation CAR-T cells had a limited lifespan and therapeutic impact because optimum T cell activation requires simultaneous signaling through TCR-CD3 and CD28 [35].

#### Second-generation CAR-T cells

To further enhance their cellular architecture, second-generation CAR-T cells add a co-stimulatory signaling domain, like CD28 or 4-1BB (CD137). In 1998, Finney and colleagues found that constructs with proximal CD28 signaling domains and membrane-distal zeta chains were more efficiently expressed in Jurkat and could mediate up to 20-fold IL-2 production upon stimulation with solid-phase Ag [36]. Subsequently, the team used CD28, inducible co-stimulators, CD134 or CD137 signaling domains in tandem with the TCR ζ chain signaling domain to generate human CD33-specific chimeric receptors and transfected them into resting human primary T cells by electroporation. The results showed that the addition of the above co-stimulatory signaling domains could enhance the levels of IL-2, IFN-γ, TNF-α and GM-CSF cytokine production induced by specific antigens, and could promote the survival and proliferation of resting primary T cells in the absence of any exogenous factors [37]. Nowadays, all clinically approved CARs are of 2nd generation.

#### Third-generation CAR-T cells

By incorporating 4-1BB, the longevity of central memory T cells is prolonged, and CD28 stimulates the development of effector memory T cells, which in turn enhances the persistence of CAR-T cells within patients [38, 39]. An extra co-stimulatory domain is present in third-generation CAR-T cells, which increases antitumor activity [40]. The effectiveness of these treatments, however, differs from patient to patient [41, 42].

#### Fourth-generation CAR-T cells

The fourth-generation CAR-T cells, also known armored CAR-T cells, including three types: T cells redirected to universal cytokine-mediated killing (TRUCK) CAR-T cells, antibody-secreting CAR-T cells, and cytokine-modulating CAR-T cells [43]. TRUCK are composed of a pair of second-generation CARs and cytokines such as IL-12 and IL-18. They can induce cytokine expression after recognizing target antigens, thereby regulating the local immune microenvironment and recruiting effector immune cells [44]. They can also regulate the cytokine environment in the tumor microenvironment, thereby enhancing the anti-tumor effect of CAR-T cells and immune cells [43]. This configuration improves T cell function in certain tissues by regulating the targeted production and release of bioactive substances such as cytokines [45].

#### Fifth-generation CAR-T cells

**Fig. 2:**
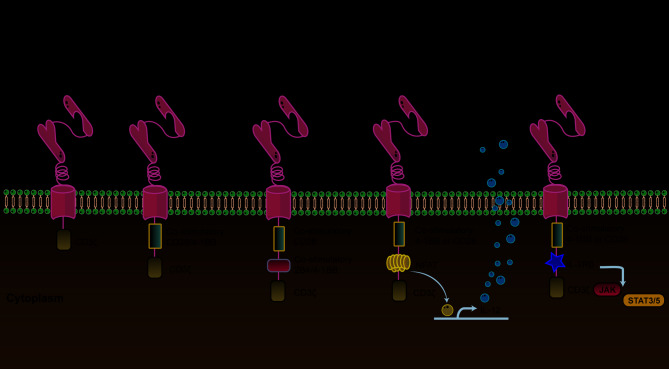
First generation to fifth generation CAR-T. The first generation of CAR-T contains an extracellular antigen recognition domain and an intracellular CD3ζ signaling domain. The second generation of CAR-T cells added a signaling domain of a co-stimulatory receptor (such as 4-1BB/CD137 or CD28) to the original structure. On this basis, a second co-stimulatory signaling domain was added to form the third generation of CAR-T cells. The fourth generation of CAR-T is based on the second generation of cells and adds cytokines (such as IL-12, IL-18). The fifth generation of CAR-T cells is also transformed from the second generation of CAR and is a new type of CAR structure that is still under study. They contain a truncated IL-2 receptor β chain domain. This domain has a binding site for the transcription factor STAT3, and the antigen-specific activation of the receptor can be achieved through the CD3ζ domain, CD28 domain and JAK-STAT3/5 signaling pathway

.The addition of a shortened IL-2 receptor β chain domain that interacts with the transcription factor STAT3 improves T cell activation and proliferation in the fifth-generation CAR-T cells, which are derived from the second-generation models (Fig. 2). All three activation signals—TCR, co-stimulatory, and cytokine—can be activated at the same time because of this [46].

#### Other CAR structures

To refine CAR-T cell sensitivity, specificity, antigen recognition, and safety, a variety of CAR formats have been introduced, including bidirectional Tandem CARs (TanCARs), dual-targeted CARs, trivalent tandem CARs, and inhibitory CARs (iCARs) [47–51]. Logic gating provides a new engineering strategy for the design of novel CAR structures. TanCARs achieve multi-point targeting by expressing a single bivalent CAR containing tandem domains, while dual-targeted CARs achieve dual-point targeting by co-expressing two independent CARs. This phenomenon that any of the multiple antigens can be used as a target is defined as an OR-gate in Boolean terminology, which has advantages in mitigating antigen escape. When encountering cells other than target cells, the gating form that uses the action of iCARs to prevent CAR-T cells from taking effect is called “NOT-gate“ [52]. “AND-gate” requires CAR-T cells to recognize two target antigens on the cell surface at the same time for activation [53]. This strategy can be used in systems using synthetic Notch receptors, that is, releasing transcription factors on a certain binding antigen, thereby driving the expression of another antigen-specific CAR [54].

In addition, Muliaditan et al. designed a parallel CAR (pCAR) that can co-express the second-generation CAR (CD28 + CD3ζ) and a chimeric costimulatory receptor containing 4-1BB, and proved that pCAR can well utilize synergy and tumor-dependent costimulation to resist T cell exhaustion and aging [55]. Studies have shown that truncated extracellular T-cell immunoglobulin mucin domain molecule 3 (TIM-3) can fuse with the CD28 transmembrane domain and cytoplasmic domain, and the formed TIM-3/CD28 (T3/28) chimera can serve as a chimeric switch receptor connected to the second-generation CAR, thereby significantly prolonging the persistence of CAR-T cells [56]. To further enhance the comprehensiveness of CAR, Cho et al. proposed a split, universal, and programmable (SUPRA) CAR system. The system consists of a universal receptor (zipCAR) expressed on T cells and a tumor-targeted scFv adapter protein (zipFv). The zipCAR universal receptor is produced by the fusion of an intracellular signal transduction domain and a leucine zipper as an extracellular domain, while the zipFv adapter molecule is produced by the fusion of a homologous leucine zipper and scFv. It was finally confirmed that the structure can achieve dual induction, which can enhance flexibility, specificity and controllability [57]. However, it still faces challenges such as tumor recurrence and drug resistance, and the influencing factors include antigen escape, CAR-T cell exhaustion and immunosuppressive microenvironment [58]. Research into the structure and function of CARs is ongoing and evolving.

### Clinical application of CAR-T cells

CAR immunotherapy was initially suggested by Eshhar in 1993 [59]. In 1996, June’s team embarked on foundational experiments with HIV patients to probe the clinical efficacy of CAR-T cells [60]. By 2010, Rosenberg and colleagues had administered the second-generation CAR-T cells to a patient suffering from refractory Follicular lymphoma (FL) [61]. Immunohistochemical staining of bone marrow biopsy tissue 36 weeks after treatment confirmed the in vivo specificity of the anti-CD19 CAR-T cells. A slew of new clinical studies were launched as a result of this research [31].

Tisagenlecleucel (Kymriah), whose official name was CTL019, was the first CAR-T treatment to get approval from the FDA [62]. This treatment put a child in remission from acute lymphoblastic leukemia (ALL) in just one month in 2012. A new treatment approach for relapsed and refractory (R/R) ALL was introduced when another child with ALL achieved remission after receiving a comparable course of treatment [63]. There has been a dramatic increase in the number of clinical trials utilizing CAR-T cell immunotherapy since then, with around 100 new trials being registered per year [62].

Clinical CAR-T therapy approvals were initiated in 2017 by the FDA. Regulatory bodies such as the European Medicines Agency (EMA) and Health Canada, continue to refine guidelines for the proper utilization and advancement of these treatments [64, 65]. 13 CAR-T cell therapy drugs have received global approval (Table 1), with six authorized for marketing in China by the National Medical Products Administration (NMPA). These include Akirencel Injection, Rekiorencel Injection, Ikiorencel Injection, Nakiorencel Injection, Zevokiorencel Injection, and Cidakiorencel Injection [66]. Notably, Cidakiorencel Injection (commercially known as Carvedi), introduced in the Chinese market in 2024, was approved by the FDA as early as 2022 [67]. This product represents a CAR-T cell therapy independently developed in China and subsequently made available in the United States. In addition, Talicabtagene autoleucel (NexCAR19™) was approved in India in October 2023 for the treatment of relapsed/refractory B-cell lymphoma (R/R BCL) and relapsed/refractory B-cell acute lymphoblastic leukemia (R/R B-ALL), and is the first CAR-T therapy approved in India [68].

**Table 1:** Summary of information on CAR-T products approved for marketing worldwide

Drug name	Generic name	Target	Indications	Year of Approval	Available regions	Manufacturer
Tisagenlecleucel	Kymriah	CD19	R/R BCP-ALL; R/R LBCL	2017	United States, European Union, Japan	Novartis
Axicabtagene ciloleucel	Yescarta	CD19	R/R LBCL; R/R FL	2017	United States, European Union, Japan	Gilead/Kate Biopharmaceuticals
Brexucabtagene autoleucel	Tecartus	CD19	R/R MCL; R/R BCP-ALL	2020	United States, European Union	Gilead/Kate Biopharmaceuticals
Lisocabtagene maraleucel	Breyanzi	CD19	R/R LBCL	2021	United States, European Union, Japan	Bristol-Myers Squibb
Idecabtagene vicleucel	Abecma	BCMA	R/R MM	2021	United States, European Union, Japan	Bristol-Myers Squibb/Bluebird Biopharmaceuticals
Akilocell injection	Yikaida	CD19	R/R LBCL	2021	China	Fosun Kate
Rekiolcell injection	Benoda	CD19	R/R LBCL; R/R FL	2021	China	Innovent Biologics
Ciltacabtagene autoleucel	Carvykti*	BCMA	R/R MM	2022	United States, European Union	Legend Biopharmaceuticals
Talicabtagene autoleucel	NexCAR19™	CD19	R/R BCL; R/R B-ALL	2023	India	Indian Institute of Technology, Bombay and Immunoadoptive Cell Therapy
Ikiolcell injection	Fukusu	BCMA	R/R MM	2023	China	Caribou Bio
Nakiolcell injection	Yuanruida	CD19	R/R B-ALL	2023	China	Reindeer Biopharmaceuticals
Zevokiolcell injection	Saikaize	BCMA	R/R MM	2024	China	Cogen Pharmaceuticals
Cidakiolcell injection	Kaweidi*	BCMA	R/R MM	2024	China	Legend Biotech
Aucatzyl	obecabtagene autoleucel, obe-cel	CD19	R/R B-ALL	2024	United States	Autolus Therapeutics

Note: Data are from FDA, EMA, Japan PMDA, India CDSCO, China NMPA websites. *Indicates that the two are the same product

However, most of the currently approved CAR-T products are used to treat hematological malignancies. Unlike hematological malignancies that do not form tissue structures, solid tumors have special histopathological characteristics, such as rich vascular tissue, wide blood vessel walls, poor structural integrity, lack of lymphatic return, and selective extravasation and retention of macromolecular drugs. These characteristics constitute a unique “enhanced permeability and retention effect (EPR effect)”, which will affect the therapeutic effect of drugs [69]. Due to the complexity of solid tumors and their locations in the human body, CAR-T cells face many obstacles in the application of solid tumor patients.Solid tumors commonly lack the chemokines required to draw in T cells that possess the matching chemokine receptors. This results in CAR-T cells struggling to navigate to and invade the tumor location, restricting their potential to carry out effective anti-tumor effects. On the other hand, solid tumors have tumor-associated fibroblasts and myeloid cells that form extracellular matrix, preventing T cells from continuously contacting tumor cells [27]. The immunosuppressive tumor microenvironment present in solid tumors can also inhibit the killing ability of T cells, making CAR-T cell treatment more difficult [70]. In addition, the heterogeneity of tumor cells makes CAR-T cells have inconsistent homing ability in the treatment of solid tumors, which limits the ability of T cells to circulate in the body and to find and bind targets in tumor tissues [21]. Immune-related adverse events, such as CRS, killing of normal cells or organs, also occurred during treatment [71].CAR-T cell therapy faces severe challenges in the three important stages of tumor detection, tumor entry, and sustained survival in tumors, and new strategies need to be found. Although clinical trials conducted in patients with solid tumors have provided some data on feasibility and safety, the relationship between the number of CAR-T cells in the blood and the number of cells actually present in the tumor site remains unclear due to the many variables involved in the clinical trial process. At present, the reasons for the failure of CAR-T cell therapy in clinical trials have not been fully clarified. Some of the reasons may be the low efficiency of CAR-T cells entering tumors, insufficient accuracy in identifying tumors, and limited cell persistence [72]. In 2006, Lamers et al. initiated a phase I clinical trial of CAR-T cell immunotherapy targeting carbonic anhydrase IX (CAIX) for the treatment of clear cell renal cell carcinoma, which verified the feasibility of CAIX as a target for solid tumor treatment and was one of the early explorations of CAR-T cells for solid tumor treatment. However, due to the low expression level of CAIX in the liver, some patients experienced dose-related liver toxicity, indicating that the expression background of the target in normal tissues should be considered when selecting the target [73, 74]. In a clinical trial evaluating the safety of adoptive immunotherapy for metastatic ovarian cancer using autologous CAR-T cells targeting the ovarian cancer-associated antigen alpha-folate receptor (FR), researchers found through PCR analysis that genetically modified T cells were present in large numbers in the circulation in the first two days after transfer, but the number of cells quickly decreased, and no cells were detected in most patients one month later, indicating that the injected CAR-T cells had low effectiveness in entering the tumor, this study used a first-generation CAR structure containing the CD3ζ signaling domain to treat patients with recurrent ovarian cancer. It was the first publicly published clinical trial targeting FR to treat solid tumors, laying the foundation for subsequent FR-targeted CAR-T cell immunotherapy [35]. In another clinical trial for advanced or recurrent head and neck squamous cell carcinoma, researchers found that after delivering up to 1 billion CAR-T cells to the tumor, the cells did not leak into the circulation, which was beneficial for reducing tumor toxicity. However, it was subsequently monitored that the radioactive tracer prepared by CAR-T cells only stayed at the injection site for 48 h, indicating that the cell’s transport efficiency was low and the treatment effect on metastatic tumors was poor [75]. On the other hand, CAR-T cells may be mistakenly directed to lymphoid tissues far away from the tumor site in vivo. CD8 + T cells that have been exposed to mouse antigens and subsequently cultured in the presence of interleukin-15 (CD8IL-15) exhibit phenotypic and functional characteristics akin to those of central memory cells. Conversely, CD8 + T cells that have undergone antigen priming and are cultured with interleukin-2 (CD8IL-2) differentiate into cytotoxic effector cells [76, 77]. However, it is worth noting that Narayan et al. reported the results of a phase 1 clinical trial of CAR-T cells for the treatment of patients with castration-resistant prostate cancer (NCT: 03089203). These CAR-T cells are equipped with a dominant negative TGFβ receptor. The study found that CAR-T cell exhaustion during treatment was associated with the upregulation of several inhibitory molecules within the tumor microenvironment (TME). These findings highlight the immunosuppressive nature of the TME and suggest that overcoming T cell exhaustion is crucial for improving the efficacy and safety of CAR-T cell therapy in solid tumors [78]. However, compared with the number of CAR-T cells in the treatment of B-ALL patients, the number of intratumoral CAR-T cells identified in this study was relatively small and had limited persistence [72]. It is necessary to conduct multiple verifications of safety and efficacy in large, prospective, randomized studies in the future, and achieve the purpose of treating solid tumors by improving efficacy and optimizing the treatment window.

The Clinical Trials Registry has 1,584 trials utilizing CAR-T cell immunotherapy for cancer as of November 30, 2024. The trials were found by searching for “CAR-T” and “cancer.” Only three of these trials have progressed to Phase IV, while the majority are in Early Phase I and Phase I (75.4%). The research subjects were mainly adults aged 18 to 64 years old. Hematologic malignancies dominate the studies, with solid tumors also receiving significant attention. Recently, researchers have begun focusing on patients with refractory autoimmune diseases undergoing specific immunotherapies. Currently, there are seven registered clinical trials approved by FDA in this area, though preliminary results have yet to be reported. Of these, five address myasthenia gravis, and one target systemic lupus erythematosus. Out of 64 clinical trials that have published results, excluding one that involved radiotherapy and one drug (TAK-981) as an intervention, the principal information from the remaining 62 clinical studies (Supplementary Table).

### Adverse reactions of CAR-T cell therapy

**Fig. 3:**
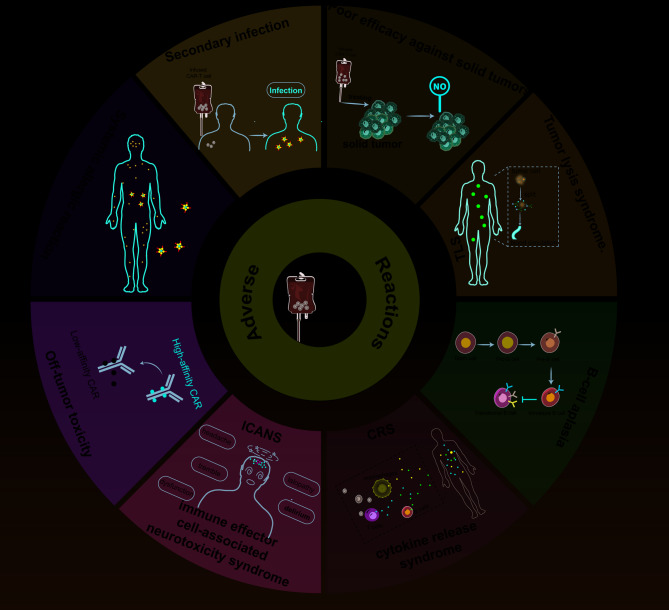
Current Adverse Reactions for CAR-T cell therapy. Adverse reactions to CAR-T cell therapy include CRS, ICANS, off-target damage, systemic allergic reactions, secondary infections, poorly treating in solid tumor, tumor lysis syndrome, and B cell aplasia

CAR-T cell immunotherapy has demonstrated effectiveness in targeting diverse cell surface molecules [79] and is extensively used in hematological malignancies, while also being explored for its potential in autoimmune diseases, viral infections, and cardiac fibrosis and solid tumors [72, 80]. Despite its efficacy, serious adverse reactions have emerged in completed clinical trials [31]. As far as adverse reactions go, CRS is by far the most prevalent, with a prevalence of 93% [81]. The second most common side effect is immune effector cell-associated neurotoxicity syndrome (ICANS). Neurotoxic side effects, off-target effects, systemic allergies, coagulopathies, B-cell aplasia, hemophagocytic lymphohistiocytosis [82], and cytopenia are other potential concerns (Fig. 3) [83].

Activated immune cells, including T cells, and so on, NK cells, and endothelium cells release inflammatory cytokines, which often trigger a systemic inflammatory response and lead to CRS [84]. It typically shows up within a week of infusion and peaks a week or two later. Sattva et al. demonstrated that including CD28 in the CAR construct reduces the onset time of CRS in patients undergoing CAR cell therapy [85].

Neurotoxic symptoms induced by activated T cells and other immune cells can manifest simultaneously or consecutively with CRS to form ICANS. Symptoms of ICANS include headaches, tremors, speech disorders, delirium, impaired consciousness, and focal functional impairments, with delirium being the most prevalent, affecting 66% of ICANS cases [86]. Reports indicate that between 21.7 and 37.2% of patients with hematological malignancies develop ICANS during treatment [83]. The occurrence of ICANS can be mitigated through proactive measures to prevent and control CRS.

The failure of CAR-T cell therapy is mainly related to the functions and properties of co-stimulatory domains in the CAR, the initial T cell phenotype (CD4 + versus CD8+), and inherent T cell quality [87]. The off-target effect is another important cause of failure and another important cause of failure and harmful consequences of CAR-T cell therapy, which occur when the therapy attacks healthy cells expressing the target antigen. Enhancing the specificity of immune cells is crucial for protecting normal cells from damage [83]. In addition, because CAR-T cell therapy suppresses bone marrow, it increases the risk of bacterial and viral infections, which usually manifest during the first 30 days after infusion [88]. Additionally, coagulation disturbances can negatively affect the quality of life for patients. These disturbances can manifest between 6 and 20 days post-infusion and include elevated fibrinogen degradation products, decreased fibrinogen levels, prolonged activated partial thromboplastin and prothrombin times, and increased D-dimer levels [89]. Considering the negative responses linked to CAR-T cell immunotherapy and its limited success in targeting solid tumors, there is significant enthusiasm for the advancement of alternative CAR-modified immune cell therapies aimed at improving treatment results and enhancing the quality of life for patients. In the pursuit of enhancing the detection and elimination of tumor cells, various immune cell types such as γδ T cells [90], NKT cells [43], NK cells [91], and macrophages [15] are being engineered to express CARs.

## CAR-NK therapy

### Origin of NK cells

NK cells, a type of lymphocyte, are primarily located in peripheral lymphoid organs and the circulatory system. As key components of the body’s innate immune system, they play a crucial role in defending against pathogens and maintaining immune integrity [92]. Bypassing the need for earlier antigen presentation or activation, NK cells can detect and destroy virus-infected cells independently of the MHC [93, 94]. The potential origins of CAR-NK cells include human embryonic stem cells, peripheral blood, umbilical cord blood, induced pluripotent stem cells (iPSCs), and NK-92 cell lines [95–98]. Originating from a non-Hodgkin lymphoma patient, the NK-92 cell line is remarkable for regulating immune responses through contact with macrophages and for its abundant production of chemokines and cytokines.

### Advantages of CAR-NK cells

**Fig. 4:**
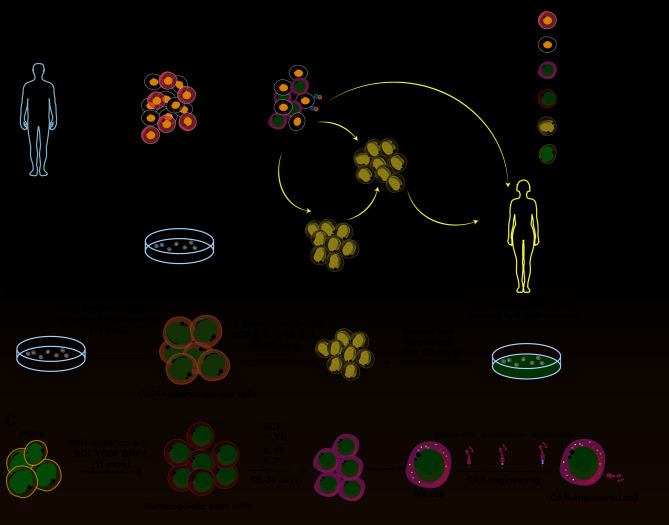
Schematic diagram of the CAR-NK cell/immunotherapy workflow. **A**-**C**) NK cells are harvested from multiple sources (**A**), activated with IL-2 (**B**), and subsequently transduced with a construct encoding the CAR (**C**)

CAR-NK cells can contain the CD3ζ intracellular domain [99], or have the CD3ζ and co-stimulatory domain 4-1BB identical to second-generation CAR-T cells [100].Research, including that of Li et al., indicates that CAR-NK cells display significant antigen-specific cytotoxicity [101]. These cells are believed to have the NKG2D transmembrane domain, the 2B4 co-stimulatory domain, and the CD3ζ signaling domain. Severe graft-versus-host disease (GVHD) prevents allogeneic CAR-T cell development by necessitating autologous T cells [102]. CAR-NK therapy avoids these complications due to its independence from MHC activation and lower GVHD risk [103]. This facilitates using widely available sources like umbilical cord blood and iPSCs for CAR-NK cell production, enabling large-scale manufacturing and readily available treatments (Fig. 4). Additionally, CAR-NK cells can reduce harmful side effects by targeting tumor cells through non-CAR-dependent pathways, which include both inhibitory and stimulatory receptors as well as antibody-dependent cytotoxic effects mediated by CD16 [104]. In tumor immunotherapy, NK cells are currently used as allogeneic products, which confirms their promise as a CAR-engineered immune cell type.

### Clinical application of CAR-NK cells

Research into CAR-NK therapy is in its infancy, and although there has been an uptick in the number of clinical trials using CAR-NK cells, the efficacy and safety need to be further confirmed. Clinical trials mainly aim to treat hematologic cancers (CD19, CD33, BCMA, and CD22) and solid tumors (NKG2D ligand, PD-L1, ROBO1, and 5T4) [11]. No CAR-NK cell immunotherapy clinical trials have progressed to phase 3 or 4, and the less than a hundred that have been registered on Clinical Trials.gov so far have all been in the early stages. To determine whether CAR-NK cells are safe and effective in treating cancer, these trials are being conducted. Only twenty-eight trials are now focusing on solid tumors, whereas the majority are investigating the anti-tumor properties of CAR-NK cells in hematological malignancies. Out of the two clinical investigations that were conducted, only one has been published thus far (Table 2).Liu et al. utilized anti-CD19 CAR-NK cells from cord blood to treat 11 patients with relapsed/refractory CD19 + non-Hodgkin’s lymphoma or chronic lymphocytic leukemia (CLL). Within two weeks of treatment, seven patients reached full remission [105]. The safety and efficacy of CAR-NK cell therapy in clinical applications were highlighted by the fact that CAR-NK cell proliferation and extended-expression were maintained for over 12 months after infusion, without any reports of CRS, neurotoxicity, or GVHD. The effectiveness and safety of CAR-NK cell therapy will need to be explored in combination with the results of more clinical trials.

**Table 2:** Clinical trials of CAR-NK cells have been completed

	NCT Number	Study Title	Targeted antigen	Study Status	Study Results	Conditions	Age	Phases	Enrollment	Start Date	Completion Date	Results First Posted	Locations
1	NCT05563545	Anti-CD19 CAR-Engineered NK Cells in the Treatment of Relapsed/Refractory Acute Lymphoblastic Leukemia	CD19	Complted	NO	ALL	Child, Adult, Older adult	I	2	July 21,2022	November 25,2022	/	China
2	NCT03056339	Umbilical & Cord Blood (CB) Derived CAR-Engineered NK Cells for B Lymphoid Malignancies	CD19	Complted	YES	B-Lymphoid Malignancies; ALL; Chronic Lymphocytic Leukemia; Non-hodgkin’s Lymphoma	Child, Adult, Older adult	I, II	49	June 21,2017	March 6,2023	March 25,2024	United States

### Challenges of CAR-NK cell therapy

Studies show that CAR-NK cell treatment has a lot of promise, but there are a lot of obstacles that make it hard to use in real-world circumstances. First, there is a limit to how long CAR-NK cells can stay in the body. Due to the limited quantity of NK cells that can be obtained from a single donor and their short duration in the body, which is usually about 14 days, patients often need to undergo multiple infusions during their treatment [106, 107]. Second, culturing NK cells in vitro is problematic. Although NK cell lines are cost-effective and possess extensive proliferative capacity, their malignant derivation mandates pre-infusion irradiation, which may reduce their longevity and therapeutic impact in vivo [11]. Moreover, gene transduction represents a formidable obstacle in CAR-NK cell therapy. Viral vectors, especially lentiviral and retroviral, are primarily used for the stable integration of CAR into the genome. Statin therapy enhances lentiviral transduction effectiveness in NK cells by upregulating low-density lipoprotein receptors, according to research by Gong et al., without reducing their cytotoxic capacities [108]. Although lentiviral vectors are effective in CAR-T cell treatment, their transfection efficiency in NK cells is often lower, hovering around 20% [109]. Studies have suggested that CAR-NK cells have lower cytotoxicity compared with CAR-T cells in vitro, but the anti-cancer activity of autologous CAR-T cells is superior to that of allogeneic CAR-NK cells in vivo, and the ability of CAR-NK cells to produce IFN-γ is sharply reduced. Therefore, methods to increase IFN-γ production in CAR-NK cells may be needed to enhance their anti-cancer activity [110]. It is worth mentioning that Zu et al. screened a membrane-proximal domain VHH targeting CD5 and combined it with engineered NK cells. Subsequently, CAR-NK cells containing 12 C nanobodies were obtained by CD5-CAR lentiviral transduction, and these cells showed killing properties against T cell malignancies both in vitro and in vivo, providing a promising treatment method for T cell malignancies [111]. In conclusion, CAR-NK cell treatment holds great promise as the next ACT step beyond CAR-T cells. To overcome the existing obstacles of CAR-NK cell therapy, three main aspects can be taken: optimizing the distance between the CAR epitope and the NK cell surface to enhance its effect; seeking more effective gene transfer methods; stimulating NK cells with cytokines or ICIs; inducing memory-like NK cells (ML NK) [112]. However, this therapy still faces problems such as lack of specific antigens, inability to develop viable and functional ready-made cell products on a large scale, and possible NK cell exhaustion [113]. To completely ascertain the effectiveness, safety, and side effects of CAR-NK therapy on patients, extensive clinical data is necessary.

## CAR-M cell therapy

**Fig. 5:**
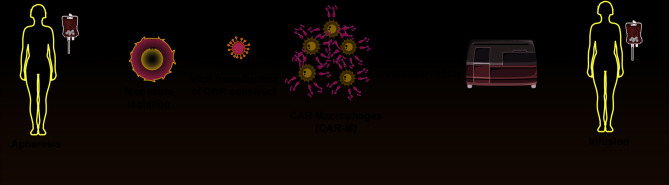
Schematic diagram of CAR-M therapy. Macrophages are first separated from other blood components in the patient’s blood. The separated macrophages are then transduced with a viral vector carrying the CAR construct. Once the macrophages are equipped with the antigen-specific CAR, they are then infused back into the patient

Although CAR-NK cells offer several advantages over CAR-T cells, they face challenges within the tumor microenvironment (TME), where NK cells are not the predominant immune cell type. The TME’s immunosuppressive nature can inhibit NK cell activity. Experiments involving NK cells transfected with a retroviral vector encoding CAR-CD19, IL-15, and a suicide gene for inducible caspase-9 have shown that the transfected NK cells can enhance in vivo proliferation and persistence, thereby improving antitumor activity and extending survival in mouse models [114]. It is unclear, however, if these results apply to other disorders in general since there has been no reporting of more than one phase I clinical trial [105]. The development of CAR technologies initially focused on T cells and NK cells before expanding to include macrophages (Fig. 5). This evolution reflects the ongoing advancement and intensification of research in cellular immunotherapy [115]. Macrophages, which are recruited to the TME by tumor-derived chemokines, can be differentiated into two primary types: M1 macrophages, which exhibit anti-tumor and pro-inflammatory characteristics, and M2 macrophages, which regulate immune responses and facilitate angiogenesis [116]. Klichinsky et al. engineered CAR-M cells targeting mesothelin and human epidermal growth factor receptor-2 (HER-2), noting a marked reduction in tumor burden in mice following the infusion of these cells [117]. This approach surpasses certain limits of conventional CAR-T and CAR-NK therapies and presents substantial benefits within the immunosuppressive TME. It is quickly becoming another exciting alternative treatment option (Table 3) [15, 118]. Currently, the mechanisms by which CAR-M cells exert their effects in TME include: converting to the M1 state during the activation process of antigen recognition to exert anti-tumor effects, remodeling TME by releasing inflammatory cytokines, exerting tumor phagocytosis, activating transcription factors, and infiltrating tumor cells [119].

**Table 3:** Comparison of CAR-T, CAR-NK, and CAR-M cell therapies

	CAR-T Cells	CAR-NK Cells	CAR-M Cells
Cell source	Autologous or MHC-matched allogeneic	Autologous, non-MHC-matched allogeneic or NK cell lines	Autologous. Preclinical studies use iPSCs and cell lines
In vitro expansion	Yes	Yes for autologous NK cells. The rest of the cells are pre-amplified before transduction	Autologous macrophages can. iPSCs and cell lines are pre-amplified before transduction
Cytotoxicity mechanism	CAR-dependent cell killing	CAR-dependent and NK cell-mediated CAR-independent cell killing	CAR-dependent macrophage-mediated phagocytosis; macrophage-mediated TME changes; macrophages as antigen-presenting cells stimulate immune responses
CRS	Common and severe	Uncommon	No clinical data
Advantages	Highly specific in the treatment of hematological malignancies; rapid response	Broad-spectrum anti-tumor function, less likely to cause CRS and neurotoxicity	Recognizes targeted specific tumor antigens; immunosuppressive TME, with the potential for sustained anti-tumor immunity.
Limitations	Immune barriers such as T cell exhaustion, antigen loss variants, and immunosuppressive TME	Low transfection efficiency, not easy to expand in vitro	Low transfection efficiency
Clinical Research Status	It has been approved for clinical treatment. Its efficacy has been confirmed in hematological cancers, and research is currently focused on diseases such as solid tumors	The most studied and extensively studied immune cell besides T cells, but with no approved therapies	Preclinical experiments have achieved good results and are an effective method for immunotherapy of solid tumors, but no clinical research results are available

### Advantages of CAR-M cell therapy

The structural components of CAR-M cells include an intracellular signaling domain, a transmembrane domain, a hinge region, and an extracellular antigen-binding domain. To improve their phagocytosis and tumor antigen presentation capabilities, these cells are genetically engineered with specialized CARs [120]. In CAR-M cell research, CD3ζ, CD147, Megf10, and FcRγ are the most commonly utilized signaling domains [11]. A novel CAR that inserts the intracellular domains of Megf10 or FcRγ into the J774A.1 mouse macrophage cell line using a lentiviral vector was pioneered by Morrissey et al. With this breakthrough, “CARs for Phagocytosis (CAR-Ps)” were first conceived, marking a significant step forward in CAR technology [121].

When it comes to treating solid tumors, CAR-M cells have clear benefits over CAR-T cells. First and foremost, they enhance the TME by facilitating communication between different types of immune cells. A thorough immune assault on tumors is triggered by the antigen-presenting characteristics of CAR-M cells, which promote communication regarding target cells throughout the immune system. In addition, CAR-M cells can induce M2 macrophages to polarize towards the M1 phenotype and secrete pro-inflammatory cytokines [120]. In addition to enhancing T cell-mediated tumor death through enabling antigen presentation and promoting a pro-inflammatory TME, studies indicate that CAR-M cells exhibit specific cytotoxicity [117]. Furthermore, CAR-M cell therapy might be considered a safer alternative to other genetically engineered cellular therapies. The use of allogeneic macrophages helps avoid the risks associated with GVHD and bypasses the need for MHC compatibility. CAR-M cells have a limited circulation time, reducing potential complications from the extended activity of modified cells [11]. Notably, upon activation through HER2 antigen recognition and subsequent CD147 signaling, CAR-M cells have demonstrated the ability to substantially reduce cytokine levels associated with CRS, including TNF-α and IL-6, thereby lowering the risk of severe or fatal CRS [122].

### Preclinical research of CAR-M cell therapy

Table 4 shows that there have only been four CAR-M cell clinical trials filed on ClinicalTrials.gov thus far. First, CT-0508 targets HER-2 in relapsed or refractory solid tumors using CAR-M cells generated from iPSCs modified with the Ad5f35 chimeric adenovirus vector. Highlighting the critical need to advance CAR-M therapeutics, Carisma’s groundbreaking medicine was granted fast-track designation by the FDA in September 2021 [123]. The critical need to progress CAR-M treatments was further highlighted in September 2021 when the FDA granted fast-track status to CT-0508. Using tumor organoids from one hundred patients, the second trial (NCT05007379) observes how CAR-M cells combat tumors [124, 125]. Uncertainty surrounds the current status of the third trial, which began in China in 2024 and aims to evaluate CAR-M cell treatment for advanced HER-2-positive gastric cancer patients’ safety and efficacy [126]. There is also a single-center, single-arm, dose-escalating, exploratory clinical trial (NCT06562647) designed to test the safety, tolerability, pharmacokinetics and preliminary efficacy of SY001, a CAR-M cell therapy product produced by Sungene Biotech Co., Ltd., in patients with advanced mesenchymal-positive solid tumors. This is the first study of a CAR-M cell product initiated by Chinese researchers. Currently, the clinical trial is actively recruiting patients [127]. Additional clinical data is needed to confirm the therapeutic efficacy and clinical viability of CAR-M cell therapy, which is still in its emerging stage due to the small number of clinical trials.

**Table 4:** Clinical trials of CAR-M cells

	NCT Number	Study Title	Targeted antigen	Study Status	Study Results	Conditions	Interventions	Sex	Age	Phases	Enrollment	Study Type
1	NCT04660929	CAR-macrophages for the Treatment of HER2 Overexpressing Solid Tumors	HER2	Active not recruiting	NO	Adenocarcinoma; Biliary Tract Cancer; Bladder Cancer; Breast Cancer; Hepatocellular Carcinoma; Lung Cancer; Ovarian Epithelial Carcinoma; Small Cell Carcinoma; Squamous Carcinoma; Transitional CellCarcinoma; Colorectal Cancer; Esophagogastric Junction Neoplasms; Stomach Neoplasms; Pancreatic Cancer; HER2-positive Solid Tumors; Prostate Cancer; Head and Neck Cancer; Endometrial Cancer	Biological: CT-0508; Biological: Pembrolizumab	Male; Female	Adult, Older adult	I	48	Interventional
2	NCT05007379	Cohort Study to Determine the Antitumor Activity of New CAR-macrophages in Breast Cancer Patients’ Derived Organoids	HER2	Unknown	NO	Breast Cancer	NA	Male; Female	Adult, Older adult	NA	100	Observational
3	NCT06224738	Human HER2-targeted Macrophages Therapy for HER2-positive Advanced Gastric Cancer With Peritoneal Metastases	HER2	Not yet recruiting	NO	Gastric Cancer	Biological: human HER2-targeted CAR-M cells	Male; Female	Adult, Older adult	Early Phase I	9	Interventional
4	NCT06562647	SY001 Targets Mesothelin in a Single-arm, Dose-increasing Setting in Subjects With Advanced Solid Tumors	Mesothelin	Recruiting	NO	Treatment Related Cancer; Ovarian Cancer	Drug: SY001	Female	Adult, Older adult	NA	2	Interventional

### Disadvantages of CAR-M cell therapy

There are a number of obstacles that CAR-M cell therapy is currently encountering in its early stages of clinical evaluation, which calls for continuous study and validation. In order to exert their lethal effects, CAR-M cells, like CAR-T cells, must traverse the intricate seven-step cancer immunity cycle [9]. Given that human macrophages form a minimal fraction of peripheral blood and lack proliferative capacity in vitro and in vivo, frequent administrations are essential to sustain adequate CAR-M cell levels for effective tumor monitoring. Moreover, research indicates that CAR-M cells predominantly accumulate in the liver rather than tumors after transiting through the lungs, adversely affecting therapeutic efficacy and heightening the risk of hepatic damage [117]. A further obstacle involves the requisite optimization of CAR-M constructs; macrophages, differing significantly in function and activation from NK and T cells, demand tailored CAR configurations to better accommodate their biological characteristics. Future investigations should aim at pinpointing appropriate intracellular domains to refine CAR architecture and enhance the conversion of primary human macrophages [121].

Overall, while CAR-M cells have demonstrated promising anti-tumor effects in preclinical studies, the safety of these treatments in clinical settings remains unconfirmed. Consequently, more extensive and detailed research is essential to fully ascertain their potential for treating cancer patients.

## Other CAR-engineering therapies

There has been a lot of focus on the immunotherapeutic possibilities of macrophages, NK cells, and T cells. Other immune cells generated with CAR technology are also being studied more and more for their potential immunotherapeutic effects. Indirectly attacking tumor cells through activating CD8 + T cells without relying on MHC, NK T (NKT) cells are a distinct population of innate T cells that take on NK cell traits after thymic maturation [128]. Furthermore, NKT cells have the ability to release cytokines as they mature, which helps to bridge the gap between the innate and adaptive immune systems and shows that they can fight tumors [129]. Adoptive therapies utilizing NKT cells have been explored in clinical settings. Heczey et al. have led the way in CAR-NKT cell research by introducing ganglioside GD2 into NKT cells through retroviral transduction, thus creating GD2-modified CAR-NKT cells and validating the potential of NKT cells in CAR-engineered cancer immunotherapy [130]. The immunosuppressive TME in solid tumors greatly hinders CAR-NKT cell activity and durability, although these therapies have demonstrated promise in treating hematological malignancies [112]. Further complicating their clinical usefulness is the variety of tumor antigens, which in turn increases the probability of antigen escape [7].

In a similar vein, γδT cells serve as a link between innate and adaptive immunity, and they are crucial for immunological protection and monitoring [131]. Gentles et al. found a strong correlation between γδT cell tumor invasion and better patient outcomes in a large gene expression investigation that included 18,000 tumor samples [132]. In 2004, Rossig’s team pioneered the expansion of peripheral blood-derived γδT cells and their modification with CARs using retroviral vectors, establishing the first CAR-γδT cells [133]. Subsequently, Beatson et al. confirmed that the presence of TGF-β1 can effectively increase the production and activity of γδT cells, and enhance their cytolytic activity, cytokine release and anti-tumor activity in some leukemia and solid tumor models [134]. Nonetheless, the diverse and suppressive TME curtails the efficacy of this strategy [135], with clinical trial results yet to be reported, leaving the clinical effectiveness of CAR-γδT cells uncertain.

Potential future cancer immunotherapies include CAR-hematopoietic stem cells, CAR-memory T cells, iPSC-derived CAR-T (iCAR-T), Cytokine-induced killer (CIK) cells [6], and CAR-Treg cells, in addition to NKT and γδT cells [7, 43, 135–139]. To determine the clinical feasibility and possible adverse effects of these techniques, which are still in the experimental phase, substantial research is required.

## Challenges and opportunities of CAR-Engineered cell immunotherapy

Although the functional durability, effectiveness and safety of CAR engineered cells are worthy of deep consideration, it is undeniable that the emergence of this immunotherapy has presents new therapeutic avenues for the treatment of tumors. Currently, all approved commercial CAR-T cell therapies use γ-retrovirus or lentivirus for genetic engineering to achieve high transduction rate and long-term stable transgene expression [140]. However, the production process faces problems such as high cost, long production time, limited production facilities, and insufficient production scale [141]. Compared with viral vectors, lipid nanoparticles (LNP)-mRNA delivery system has the advantages of no host genome integration, low cost, low toxicity, and high modifiability [142, 143]. Prazeres et al. optimized the ionizable lipid nanoparticle (LNP) formulation, which can deliver pDNA to primary human T cells more efficiently, and provide new ideas for the treatment of solid tumors and other diseases by engineering CAR-T cells to kill tumor cells specifically [141]. Bozza et al. researched DNA vectors. They confirmed that non-integrating DNA vectors with enhanced transgenic cell generation can rapidly generate CAR-T cells on a clinical scale in a closed system, and have strong anti-tumor activity both in vitro and in vivo [144]. In addition, due to the interaction between the CAR structure and the antigen, it can cause fratricide, which can be avoided by inhibiting the expression of the surface target antigen [145]. High-throughput screening methods using small interfering RNA (siRNA), short hairpin RNA (shRNA) or CRISPR screening to identify effective gene targets and potential drugs or delivery vectors are one of the strategies to advance CAR-T cell therapy [12]. The clustered regularly interspaced short palindromic repeats/associated protein 9 (CRISPR-Cas9) gene-editing technology can enhance efficacy and durability by targeted modification of genes [146], knock out genes that inhibit T cell function and regulate CAR-T cell activity in the tumor microenvironment [147], thereby improving the effectiveness, safety and accessibility of CAR-T immunotherapy [148]. In addition to gene modification, Hu et al. engineered 293T cells by introducing the scFv (anti-EpCAM)-41BB-CD3ζ expression gene into the cells, and then used a fusion agent to fuse the CAR-293T cells with Jurkat cells. The results showed that the fused CAR-T cells showed better targeting ability. This study provides a new perspective for the development of universal CAR-T cells and helps to accelerate the construction of non-genetically modified CAR-T cells [149]. Advanced methods such as single-cell RNA sequencing and artificial intelligence have also been applied to identify tumor biomarkers to improve CAR-T cell design and predict treatment response [150, 151]. On the other hand, some scholars have pointed out that PROTAC technology and OVs loaded into CAR-T cells can be used as an alternative to combined therapy, which is beneficial to reduce the adverse reactions caused by combined medication [7]. Organoids derived from in vitro three-dimensional culture systems similar to tissues or organs also provide new materials and targets for CAR-T cell therapy [87]. Biomaterials such as hydrogels, microneedles and annular spiral particles are also used to load CAR-T cells for dispersed inoculation in solid tumors, which helps to achieve precise delivery. In actual application, the strategy of dose-fractionated administration can stagger the rise in cytokine levels, reduce their peak values, and thus reduce the severity of CRS [152]. Metabolic regulation strategies have also been shown to prolong the lifespan of T cells. CAR-T cells that use oxidative phosphorylation and fatty acid oxidation have greater persistence than cells that rely on glycolysis, which affects patient prognosis. Therefore, CAR-T cells can be metabolically engineered to oxidative phosphorylation, thereby extending lifespan through epigenetic and phenotypic changes [153].

As a pioneer in CAR-M research, Carisma Therapeutics’ core product CT-0508 showed good tolerability in Phase 1 clinical trials, but its effects on cell persistence and tumor infiltration efficiency were poor. It was eventually suspended in 2024 due to a long R&D cycle and capital chain pressure. This case shows that even if the technology is innovative, it may face the risk of commercial failure if it cannot be clinically transformed in the short term. In addition, the survival window of patients with advanced cancer also requires the application of new therapies to accelerate clinical transformation efficiency.

To improve the safety and efficacy of CAR-related products in clinical applications, it is necessary to address issues such as regulation and transformation involved in the process of transitioning from preclinical research to clinical application [154]. The currently commonly used supply method for autologous CAR cell therapy is to collect samples from various hospitals and/or leukocyte apheresis sites, then transfer them to processing sites, and finally deliver them to hospitals. In this process, there are problems such as long cycles, inconsistent storage temperatures, difficult sample tracking, and short shelf life [154]. Indian researchers used the fully automated, closed, and GMP-compliant CliniMACS Prodigy^®^ system (Miltenyi Biotec) to produce clinically available CD19-targeted CAR-T cells, and the viability of the CAR-T cell products exceeded 90% in three runs, with higher transduction efficiency and consistent viability, good reproducibility and safety, and met the requirements of clinical application. Compared with manual production, this method can greatly save manpower, time and cost, and provides the possibility for the widespread application of CAR cells [155]. Recently, this result was confirmed again in a study in Thailand [156]. In addition, commercially produced CAR cell products also face challenges such as insufficient production capacity, long production cycle and high cost. To this end, Elsallab and his colleagues proposed the use of automated cell production systems and online sensors to enhance quality control and supply chain management, supplement existing supplies with cells produced at local distributed sites, ensure that patients obtain commercially available products and robustly develop future therapies, and find an appropriate balance between clinical needs and drug regulation [157]. These attempts have pointed out the direction for the development of subsequent CAR cell engineering immunotherapy.

## Conclusions

CAR-T cell therapy, emerging as the foremost rapidly evolving form of ACI, is progressively utilized in the treatment of cancer. Still, problems including the immunosuppressive TME and a lack of tumor-specific antigens have led researchers to expand CAR engineering to include other types of immune cells with anti-tumor capabilities, such as NK cells and macrophages. However, NK cell short half-life and inefficient transduction pose problems for clinical uses of CAR-NK cell treatment. Similarly, CAR-M cells are difficult to produce, and owing to macrophages’ great flexibility and restricted growth capacities, they do not work as well as intended in vivo. The therapeutic potential of CAR-NKT cells has been confirmed by studies, however, the biggest obstacle is still scaling up their in vitro expansion. For CAR-γδT cells, although no severe adverse effects have been reported thus far, their very low prevalence in the body and challenges in efficient expansion and isolation represent formidable barriers to their clinical use.

Although more and more CAR engineered cell immunotherapies are being developed, CAR-T, CAR-M and CAR-NK therapies still occupy an important position. The selection of their clinical application should consider multiple factors such as patient status, disease type and treatment purpose. CAR-T cells have achieved significant therapeutic effects in B-cell malignancies due to their good expansion ability and persistence, but their adverse reactions such as CRS and neurotoxicity limit their application. In contrast, the activation of CAR-NK cells does not depend on MHC, has lower toxic side effects and off-target effects, is suitable for the development of allogeneic universal products, and shows potential in the treatment of solid tumors and virus-related tumors. CAR-M cells, by reshaping TME and enhancing antigen presentation, make up for the shortcomings of other cell therapies that are limited in activity in TME. Therefore, in specific clinical applications, individualized cell therapy plans should be formulated based on factors such as the immune characteristics of the targeted disease, individual differences in patients, and treatment costs and accessibility to achieve the optimal balance between treatment effectiveness and safety.

Although these new technologies have offered promising alternatives for the treatment of tumors, their efficacy when used alone is difficult to determine. In this regard, combined therapy between various CAR engineered cells may be a desirable solution. In addition, enhanced immunotherapy that directly enhances or genetically modifies cells through drugs or biotechnology to improve the efficacy of ACI has also become a research hotspot. Drugs or biotechnologies that can be used to enhance the killing ability of immune cells include ICIs/antibody drugs, small molecule inhibitors, immunomodulatory factors, proteolysis-targeted chimeras, oncolytic viruses, etc. In summary, future research should focus on the development of universal cell products or the selection of the best single type or combination of CAR engineered immune cells for precise treatment and enhanced treatment according to the patient’s condition.

## Electronic supplementary material

Below is the link to the electronic supplementary material.

Supplementary Material 1

## Data Availability

No datasets were generated or analysed during the current study.

## Abbreviations

CAR: Chimeric Antigen Receptor
ICI: Immune Checkpoint Inhibitor
ACI: Adoptive Cellular Immunotherapy
CAR-T: Chimeric Antigen Receptor T cell
FDA: The U.S. Food and Drug Administration
CRS: Cytokine Release Syndrome
CAR-NK: CAR-engineered Natural Killer Cell
CAR-M: CAR-macrophage
TCR: T Cell Receptor
MHC: Major Histocompatibility Complex
scFv: Single-Chain Variable Fragment
ALL: Acute Lymphoblastic Leukemia
R/R: Relapsed and Refractory
DLBCL: Diffuse Large B Cell Lymphoma
BCP-ALL: B-cell Precursor ALL
MCL: Mantle Cell Lymphoma
MM: Multiple Myeloma
BCMA: B Cell Maturation Antigen
EMA: European Medicines Agency
NMPA: National Medical Products Administration
FR: Folate Receptor
ICANS: Immune Effector Cell-Associated Neurotoxicity Syndrome
iPSC: Induced Pluripotent Stem Cell
GVHD: Graft-Versus-Host Disease
CLL: Chronic Lymphocytic Leukemia
TME: Tumor Microenvironment
HER-2: Human Epidermal Growth Factor Receptor-2
