# Supplementary material for: Current challenges and emerging opportunities of chimeric antigen receptor-engineered cell immunotherapy
Source: Exp Hematol Oncol. 2025 Jul 2;14:92. doi: 10.1186/s40164-025-00683-y (PMC12224691; doi:10.1186/s40164-025-00683-y)
Supplement: Supplementary file 1 — Supplementary Material 1 [file 40164_2025_683_MOESM1_ESM.docx]

| Number | NCT Number  Supplementary Table Results of Clinical Trial with results of 62 Cases of Cancer Treated With CAR T Cells | Study Title | Targeted antigen | Conditions | Phases | Age(Child (birth-17); Adult (18-64);Older adult (65+)) | Enrollment | Locations | Study Status | Terminated reasons | Efficacy | Safety |
| --- | --- | --- | --- | --- | --- | --- | --- | --- | --- | --- | --- | --- |
| 1 | NCT00924287 | Gene Therapy Using Anti-Her-2 Cells to Treat Metastatic Cancer | HER-2 | Metastatic Cancer | Ⅰ; Ⅱ | Adult, Older adult | 1 | United States | Terminated | The first patient on study died | NA | SAE: 100.0%;  Other (Not Including Serious) Adverse Events: 100.0% |
| 2 | NCT00924326 | CAR T Cell Receptor Immunotherapy for Patients With B-cell Lymphoma | CD19 | Primary Mediastinal B-cell Lymphoma; Diffuse BLCL; Diffuse LBCL Transformed From FL; Mantle Cell | Ⅰ; Ⅱ | Adult, Older adult | 43 | United States | Completed | / | CR: 54.3%; PR: 8.7% | SAE: 63.0%;  Other (Not Including Serious) Adverse Events: 100.0% |
| 3 | NCT01218867 | CAR T Cell Receptor Immunotherapy Targeting VEGFR2 for Patients With Metastatic Cancer | VEGFR2 | Metastatic Cancer; Metastatic Melanoma; Renal Cancer | Ⅰ; Ⅱ | Adult, Older adult | 24 | United States | Terminated | No objective responses were observed | CR: 0.0%; PR: 4.2% | SAE: 20.8%;  Other (Not Including Serious) Adverse Events: 95.8% |
| 4 | NCT01318317 | Genetically Engineered Lymphocyte Therapy After Peripheral Blood Stem Cell Transplant in Treating Patients With High-Risk, Intermediate-Grade, B-cell Non-Hodgkin Lymphoma | CD19 | Recurrent Grade 1 FL; Recurrent Grade 2 FL; Recurrent Grade 3 FL; Recurrent Mantle Cell Lymphoma; Recurrent Non-Hodgkin's Lymphoma | Ⅰ; Ⅱ | Adult, Older adult | 8 | United States | Active not recruiting | / | PFS(at 1 year): 50.0% | SAE: 62.5%;  Other (Not Including Serious) Adverse Events: 100.0% |
| 5 | NCT01454596 | CAR T Cell Receptor Immunotherapy Targeting EGFRvIII for Patients With Malignant Gliomas Expressing EGFRvIII | EGFRvⅢ | Malignant Glioma; Glioblastoma; Brain Cancer | Ⅰ; Ⅱ | Adult, Older adult | 18 | United States | Completed | / | Median of PFS: 1.5month; OR: 0.0% | SAE: 11.1%;  Other (Not Including Serious) Adverse Events: 100.0% |
| 6 | NCT01460901 | Study of Donor Derived, Multi-virus-specific, Cytotoxic T-Lymphocytes for Relapsed/Refractory Neuroblastoma | GD2 | Neuroblastoma | Ⅰ | Child | 5 | United States | Completed | / | CR: 0.0%; Non-complete Response: 100.0% | SAE: 100.0%;  Other (Not Including Serious) Adverse Events: 66.7% |
| 7 | NCT01583686 | CAR T Cell Receptor Immunotherapy Targeting Mesothelin for Patients With Metastatic Cancer | Mesothelin | Cervical Cancer; Pancreatic Cancer; Ovarian Cancer; Mesothelioma; Lung Cancer | Ⅰ; Ⅱ | Adult, Older adult | 15 | United States | Terminated | slow/insufficient accrual | CR: 0.0%; PR: 0.0% | SAE: 33.3%;  Other (Not Including Serious) Adverse Events: 100.0% |
| 8 | NCT01593696 | Anti-CD19 White Blood Cells for Children and Young Adults With B Cell Leukemia or Lymphoma | CD19 | ALL; B Cell Lymphoma\|Leukemia; Large Cell Lymphoma; Non-hodgkin's Lymphoma | Ⅰ | Child, Adult | 53 | United States | Completed | / | CR: 59.6% | SAE: 26.4%;  Other (Not Including Serious) Adverse Events: 100.0% |
| 9 | NCT01865617 | Laboratory Treated T Cells in Treating Patients With Relapsed or Refractory Chronic Lymphocytic Leukemia, Non-Hodgkin Lymphoma, or Acute Lymphoblastic Leukemia | CD19 | CD19+ Recurrent Adult ALL; R/R Chronic Lymphocytic Leukemia; R/R Diffuse LBCL; R/R Mantle Cell Lymphoma; R/R Non-Hodgkin's Lymphoma; R/R Small Lymphocytic Lymphoma; Refractory ALL | Ⅰ; Ⅱ | Adult, Older adult | 204 | United States | Completed | / | Death(within 8 Weeks): 2.0%; DLT(30 days): 76.1%; Objective Response Rate of CR and PR: 68.0%; OS(up to 1 year): 44.2%; PFS(up to 1 year): 9.6%; | SAE: 95.9%;  Other (Not Including Serious) Adverse Events: 99.5% |
| 10 | NCT02028455 | A Pediatric and Young Adult Trial of Genetically Modified T Cells Directed Against CD19 for Relapsed/Refractory CD19+ Leukemia | CD19 | CD19+ Acute Leukemia | Ⅰ; Ⅱ | Child, Adult | 167 | United States | Active not recruiting | / | MRD Negative CR after Initial CAR T Infusion: 88.7% | SAE: 54.8%;  Other (Not Including Serious) Adverse Events: 100.0% |
| 11 | NCT02030847 | Study of Redirected Autologous T Cells Engineered to Contain Anti-CD19 Attached to TCR and 4-1BB Signaling Domains in Patients With Chemotherapy Resistant or Refractory Acute Lymphoblastic Leukemia | CD19 | R/R B-cell ALL, with no available curative treatment options | Ⅱ | Adult, Older adult | 42 | United States | Completed | / | CR(at Day 28 After CART-19 Therapy): 26.7%; CRi: 33.3%; No Response: 20.0%; Unknown: 20.0% | SAE: 100.0%; Other (Not Including Serious) Adverse Events: 100.0% |
| 12 | NCT02208362 | Genetically Modified T-cells in Treating Patients With Recurrent or Refractory Malignant Glioma | CD19 | R/R Glioblastoma; R/R Malignant Glioma | Ⅰ | Child, Adult, Older adult | 65 | United States | Active not recruiting | / | Alive(at 6 Months): 64.9% | SAE: 90.8%; Other (Not Including Serious) Adverse Events: 100.0% |
| 13 | NCT02215967 | Study of T Cells Targeting B-Cell Maturation Antigen for Previously Treated Multiple Myeloma | BMCM | Plasma-Cell Myeloma; MM | Ⅰ | Adult, Older adult | 30 | United States | Completed | / | DLT(up to 60 days): 25.0%; CR: 3.8%; PR: 15.4%; Progressive Disease: 3.8% | SAE: 50.0%; Other (Not Including Serious) Adverse Events: 100.0% |
| 14 | NCT02348216 | Study Evaluating the Safety and Efficacy of KTE-C19 in Adult Participants With Refractory Aggressive Non-Hodgkin Lymphoma | CD19 | R/R Diffuse LBCL; Transformed Follicular Lymphoma; Primary Mediastinal B-cell Lymphoma; High Grade B-cell Lymphoma | Ⅰ; Ⅱ | Adult, Older adult | 307 | United States; Canada; France; Germany; Israel; Netherlands | Completed | / | PhaseⅠOR(Assessed by Investigator Per Revised IWG Response Criteria for Malignant Lymphoma: 83.0% | SAE: 57.6%; Other (Not Including Serious) Adverse Events: 99.7% |
| 15 | NCT02445248 | Study of Efficacy and Safety of CTL019 in Adult DLBCL Patients | CD19 | Diffuse LBCL | Ⅱ | Adult, Older adult | 115 | United States; Australia; Austria; Canada; France; Germany; Italy; Japan; Netherlands; Norway | Completed | / | ORR Per Independent Review Committee (IRC) in Main Cohort(60 months): 54.5%; All Collected Deaths(Up to 61 months): 66.1% | SAE: 73.0%; Other (Not Including Serious) Adverse Events: 98.3% |
| 16 | NCT02535364 | Study Evaluating the Efficacy and Safety of JCAR015 in Adult B-cell Acute Lymphoblastic Leukemia (B-ALL) | CD19 | ALL | Ⅱ | Adult, Older adult | 82 | United States | Terminated | Safety reasons | CR or CRi: 45.5% | SAE: 60.5%; Other (Not Including Serious) Adverse Events: 100.0% |
| 17 | NCT02601313 | Study of Brexucabtagene Autoleucel (KTE-X19) in Participants With Relapsed/Refractory Mantle Cell Lymphoma (Cohort 1 and Cohort 2) | CD19 | R/R Mantle Cell Lymphoma | Ⅱ | Adult, Older adult | 105 | United States; France; Germany; Netherlands | Completed | / | OR Per the Lugano Classification According to Independent Radiology Review Committee (IRRC) in Cohort 1(Up to 2 years): 93.0%; OR Per the Lugano Classification According to Independent Radiology Review Committee (IRRC) in Cohort 2(Up to 2 years): 93.0% | SAE: 67.4%; Other (Not Including Serious) Adverse Events: 100.0% |
| 18 | NCT02614066 | A Study Evaluating the Safety and Efficacy of Brexucabtagene Autoleucel (KTE-X19) in Adult Subjects With Relapsed/Refractory B-precursor Acute Lymphoblastic Leukemia (ZUMA-3) | CD19 | R/R B-precursor ALL | Ⅰ; Ⅱ | Adult, Older adult | 125 | United States; Canada; France; Germany; Netherlands | Completed | / | CR or CRi: 70.9% | SAE: 80%; Other (Not Including Serious) Adverse Events: 100.0% |
| 19 | NCT02650999 | Pembrolizumab in Patients Failing to Respond to or Relapsing After CAR T Cell Therapy for Relapsed or Refractory Lymphomas | CD19 | CD19+ Diffuse LBCL; FL; Mantle Cell Lymphomas | Ⅰ; Ⅱ | Adult, Older adult | 12 | United States | Completed | / | DLT(3 years): 8.3%; ORR(3 months): 25.0% | SAE: 66.7%; Other (Not Including Serious) Adverse Events: 100.0% |
| 20 | NCT02659943 | T Cells Expressing a Fully-human AntiCD19 Chimeric Antigen Receptor for Treating B-cell Malignancies | CD19 | B-cell Lymphoma; Non-hodgkin's Lymphoma | Ⅰ | Adult, Older adult | 27 | United States | Completed | / | CR: 4.8% | SAE: 81.0%;Other (Not Including Serious) Adverse Events: 100.0% |
| 21 | NCT02664363 | EGFRvIII CAR T Cells for Newly-Diagnosed WHO Grade IV Malignant Glioma | EGFRvⅢ | Glioblastoma | Ⅰ | Adult, Older adult | 3 | United States | Terminated | Study funding ended | NA | SAE: 33.3%;Other (Not Including Serious) Adverse Events: 33.3% |
| 22 | NCT02706392 | Genetically Modified T-Cell Therapy in Treating Patients With Advanced ROR1+ Malignancies | ROR1 | Hematopoietic and Lymphoid Cell Neoplasm; Malignant Solid Neoplasm; Metastatic Lung Non-Small Cell Carcinoma; Metastatic Triple-Negative Breast Carcinoma; Recurrent ALL; Recurrent Mantle Cell Lymphoma; Refractory Chronic Lymphocytic Leukemia; Stage Ⅲ Lung Non-Small Cell Cancer AJCC v7; Stage IV Breast Cancer AJCC v6 and v7; Stage IV Lung Non-Small Cell Cancer AJCC v7; Unresectable Lung Non-Small Cell Carcinoma | Ⅰ | Adult, Older adult | 21 | United States | Terminated | slow accruals | CR and PR(1 year): 5.0%; PFS: 5.0%; OS: 45.0% | SAE: 60.0% Other (Not Including Serious) Adverse Events: 100.0% |
| 23 | NCT02706405 | JCAR014 and Durvalumab in Treating Patients With Relapsed or Refractory B-cell Non-Hodgkin Lymphoma | CD19 | Diffuse LBCL, Not Otherwise Specified; R/R Diffuse LBCL; R/R Primary Mediastinal (Thymic) Large B-Cell Lymphoma; Refractory High Grade B-Cell Lymphoma With MYC, BCL2, and BCL6 Rearrangements | Ⅰ | Adult, Older adult | 30 | United States | Terminated | slow accrua | CR(1 year): 35.7%; PR(1 year): 14.3%; ORR(1 year): 39.3% | SAE: 71.4%;Other (Not Including Serious) Adverse Events: 100.0% |
| 24 | NCT02761915 | A Phase I Trial of Anti-GD2 T-cells (1RG-CART) | GD2 | Relapsed or Refractory Neuroblastoma | Ⅰ | Child, Adult, Older adult | 17 | United Kingdom | Completed | / | PFS(up to 2 years): 27-122 days | SAE: 41.7%; Other (Not Including Serious) Adverse Events: 100.0% |
| 25 | NCT02794246 | CART-19 Post-ASCT for Multiple Myeloma | CD19 | MM | Ⅱ | Adult, Older adult | 6 | United States | Terminated | administrative reasons | PFS(for 2-3 years post CART-19 infusion): 16.7%; PR: 50.0% | SAE: 33.3% Other (Not Including Serious) Adverse Events: 16.7% |
| 26 | NCT02926833 | Study of Safety and Efficacy of KTE-C19 in Combination With Atezolizumab in Adults With Refractory Diffuse Large B-Cell Lymphoma (DLBCL) | CD19 | Refractory Diffuse LBCL | Ⅰ; Ⅱ | Adult, Older adult | 37 | United States | Completed | / | ORR: 90.0% | SAE: 67.6%;Other (Not Including Serious) Adverse Events: 100.0% |
| 27 | NCT02935543 | CART19 in Adult Patients With Minimal Residual Disease During Upfront Treatment for ALL | CD19 | ALL | Ⅱ | Adult, Older adult | 1 | United States | Terminated | administrative reasons | MRD<0.01%: 0.0% | SAE: 100.0%; Other (Not Including Serious) Adverse Events: 0.0% |
| 28 | NCT03019055 | Study of CAR-20/19-T Cells in Patients With Relapsed Refractory B Cell | CD19, CD20 | Non-hodgkin's Lymphoma; B-cell Lymphoma;Chronic Lymphocytic Leukemia; Small Lymphocytic Lymphoma | Ⅰ | Adult, Older adult | 26 | United States | Completed | / | OR(at day 28): 82.0%; CR(at day 28): 64.0%; PR(at day 28): 8.0% | SAE: 100.0%; Other (Not Including Serious) Adverse Events: 100.0% |
| 29 | NCT03049449 | T Cells Expressing a Fully-Human Anti-CD30 Chimeric Antigen Receptor for Treating CD30-Expressing Lymphomas | CD30 | Anaplastic Large-Cell Lymphoma; Enteropathy-Associated T-Cell Lymphoma; Diffuse LBCL; Extranodal NK-T-Cell Lymphoma; Peripheral T-Cell Lymphoma | Ⅰ | Adult, Older adult | 26 | United States | Completed | / | OS: 43.0%; Median EFS: 13 weeks | SAE: 45.5%;Other (Not Including Serious) Adverse Events: 100.0% |
| 30 | NCT03287804 | APRIL CAR T Cells (AUTO2) Targeting BCMA and TACI for the Treatment of Multiple Myeloma | BCMA, TACI | MM | Ⅰ; Ⅱ | Adult, Older adult | 12 | Netherlands; United Kingdom | Terminated | Preliminary efficacy were not sufficient to warrant further development | responses: 45.5% | SAE: 54.6%; Other (Not Including Serious) Adverse Events: 100.0% |
| 31 | NCT03288493 | P-BCMA-101 Tscm CAR-T Cells in the Treatment of Patients With Multiple Myeloma (MM) | BCMA | MM | Ⅰ; Ⅱ | Adult, Older adult | 105 | United States | Terminated | The phase II portion of the study was terminated early to focus on an Allogeneic BCMA CAR-T program. | ORR(treated with single administration during the initial dose escalation): 57.0% | SAE: 54.5%; Other (Not Including Serious) Adverse Events: 92.7% |
| 32 | NCT03289455 | CD19 /22 CAR T Cells (AUTO3) for the Treatment of B Cell Acute Lymphoblastic Leukemia (ALL) | CD19, CD22 | B-ALL; R/R Childhood ALL | Ⅰ; Ⅱ | Child, Adult | 23 | United Kingdom | Completed | / | ORR: 66.0%; CR: 17.0% | SAE: 40% Other (Not Including Serious) Adverse Events: 100.0% |
| 33 | NCT03310619 | A Safety and Efficacy Trial of JCAR017 Combinations in Subjects With Relapsed/Refractory B-cell Malignancies | CD19 | Non-hodgkin's Lymphoma; Diffuse LBCL; FL | Ⅰ; Ⅱ | Adult, Older adult | 62 | United States | Completed | / | CRR(at 6 months): 60.0% | SAE: 54.8%; Other (Not Including Serious) Adverse Events: 85.5% |
| 34 | NCT03318861 | Study to Evaluate the Safety and Efficacy of KITE-585 in Participants With Relapsed/Refractory Multiple Myeloma | BCMA | R/R Multiple Myeloma | Ⅰ | Adult, Older adult | 17 | United States | Terminated | lack of efficacy | DLT(From KITE-585 infusion until 28 days after KITE-585 infusion): 0.0% | SAE: 88.2%; Other (Not Including Serious) Adverse Events: 100.0% |
| 35 | NCT03330834 | CAR-T Cell Immunotherapy for Advanced Lung Cancer | PD-L1 | Advanced Lung Cancer | Ⅰ | Adult, Older adult | 1 | China | Terminated | Serious adverse events | CR or PR(at 2 years): 100.0% | SAE: 100.0%; Other (Not Including Serious) Adverse Events: 100.0% |
| 36 | NCT03338972 | Immunotherapy With BCMA CAR-T Cells in Treating Patients With BCMA Positive Relapsed or Refractory Multiple Myeloma | BCMA | R/R Plasma Cell Myeloma | Ⅰ | Adult, Older adult | 28 | United States | Completed | / | ORR: 100.0% | SAE: 84%; Other (Not Including Serious) Adverse Events: 100.0% |
| 37 | NCT03391466 | Study of Effectiveness of Axicabtagene Ciloleucel Compared to Standard of Care Therapy in Patients With Relapsed/Refractory Diffuse Large B Cell Lymphoma | CD19 | R/R Diffuse LBCL | Ⅲ | Adult, Older adult | 359 | United States; Australia; Austria; Belgium; Canada; France; Germany; Israel; Italy; Netherlands; Spain; Sweden; Switzerland; United Kingdom | Completed | / | CR: 65.0% | SAE: 51.2%; Other (Not Including Serious) Adverse Events: 99.4% |
| 38 | NCT03430011 | Study Evaluating the Safety and Efficacy of JCARH125 in Subjects With Relapsed and/or Refractory Multiple Myeloma | BCMA | MM | Ⅰ; Ⅱ | Adult, Older adult | 165 | United States | Completed | / | ORR(in Phase 2 and Phase 2a): 94.1% | SAE: 33.3%; Other (Not Including Serious) Adverse Events: 100.0% |
| 39 | NCT03483103 | Lisocabtagene Maraleucel (JCAR017) as Second-Line Therapy (TRANSCEND-PILOT-017006) | CD19 | Non-hodgkin's Lymphoma; Diffuse LBCL | Ⅱ | Adult, Older adult | 74 | United States; | Completed | / | OR: 80.0% | SAE: 32.9%; Other (Not Including Serious) Adverse Events: 96.7% |
| 40 | NCT03548207 | A Study of JNJ-68284528, a Chimeric Antigen Receptor T Cell (CAR-T) Therapy Directed Against B-Cell Maturation Antigen (BCMA) in Participants With Relapsed or Refractory Multiple Myeloma | BCMA | MM | Ⅰ; Ⅱ | Adult, Older adult | 126 | United States; Japan | Completed | / | ORR: 97.0%; CR: 67.0%; OS: 89.0% | SAE: 50.9%; Other (Not Including Serious) Adverse Events: 100.0% |
| 41 | NCT03568461 | Efficacy and Safety of Tisagenlecleucel in Adult Patients With Refractory or Relapsed Follicular Lymphoma | CD19 | FL | Ⅱ | Adult, Older adult | 98 | United States; Australia; Austria; Belgium; France; Germany; Italy; Japan; Netherlands; Norway; Spain; United Kingdom | Active not recruiting | / | CR: 71.0% | SAE: 43.3%; Other (Not Including Serious) Adverse Events: 96.9% |
| 42 | NCT03570892 | Tisagenlecleucel in Adult Patients With Aggressive B-cell Non-Hodgkin Lymphoma | CD19 | Non-hodgkin's Lymphoma | Ⅲ | Adult, Older adult | 331 | United States; Australia; Austria; Belgium; Brazil; China; France; Germany; Italy; Japan; Netherlands; Norway; Singapore; Spain; Switzerland; United Kingdom | Active not recruiting | / | EFS(Per Blinded Independent Review Committee (BIRC) Assessment; appro. 24 months): 3.0months | SAE: 49.1%; Other (Not Including Serious) Adverse Events: 98.5% |
| 43 | NCT03573700 | Evaluation of CD19-Specific CAR Engineered Autologous T-Cells for Treatment of Relapsed/Refractory CD19+ Acute Lymphoblastic Leukemia | CD19 | R/R ALL | Ⅰ; Ⅱ | Child, Adult | 24 | United States | Active not recruiting | / | Maximum Tolerated Dose and DLT(4 weeks post-SJCAR19 infusion): 8.3%; CRR(4 weeks post-SJCAR19 infusion): 83.3% | SAE: 37.5%; Other (Not Including Serious) Adverse Events: 100.0% |
| 44 | NCT03602612 | T Cells Expressing a Novel Fully-Human Anti-BCMA CAR for Treating Multiple Myeloma | BCMA | MM; Plasma-Cell Myeloma | Ⅰ | Adult, Older adult | 35 | United States | Active not recruiting | / | DLT(First 28 days of treatment): 3.8% | SAE: 80.8%; Other (Not Including Serious) Adverse Events: 100.0% |
| 45 | NCT03610724 | Phase II Open Label Trial to Determine Safety & Efficacy of Tisagenlecleucel in Pediatric Non-Hodgkin Lymphoma Patients | CD19 | Non-hodgkin's Lymphoma | Ⅱ | Child, Adult | 34 | United States; Australia; Austria; Canada; Denmark; Finland; France; Germany; Italy; Japan; Netherlands; Norway; Spain; United Kingdom | Completed | / | ORR as Determined by Local Investigator(6 months post-tisagenlecleucel infusion): 32.1%; Response(Post-infusion Day 28, Month 3, Month 6, Month 9, Month 12, Month 18, Month 24, and then annually until Month 48): DOR: NA, EFS: 2.1months, RFS: NA, PFS: 2.5months, OS: 10.4months | SAE: 72.7%; Other (Not Including Serious) Adverse Events: 100.0% |
| 46 | NCT03624036 | Study to Evaluate the Safety and Tolerability of Brexucabtagene Autoleucel (KTE-X19) in People With Relapsed/Refractory Chronic Lymphocytic Leukemia and Small Lymphocytic Lymphoma | CD19 | R/R Chronic Lymphocytic Leukemia; R/R Small Lymphocytic Lymphoma | Ⅰ | Adult, Older adult | 16 | United States; Italy | Terminated | Development program terminated | DLT(First infusion date of brexucabtagene autoleucel up to 28 days): 6.7% | SAE: 66.7%; Other (Not Including Serious) Adverse Events: 100.0% |
| 47 | NCT03704298 | Safety and Efficacy of Axicabtagene Ciloleucel in Combination With Utomilumab in Adults With Refractory Large B-cell Lymphoma | CD19 | R/R LBCL | Ⅰ | Adult, Older adult | 15 | United States | Terminated | Development program terminated | Phase 1: DLT(Up to 28 days): 0.0% | SAE: 58.3%; Other (Not Including Serious) Adverse Events: 100.0% |
| 48 | NCT03743246 | A Study to Evaluate the Safety and Efficacy of JCAR017 in Pediatric Subjects With Relapsed/Refractory (r/r) B-cell Acute Lymphoblastic Leukemia (B-ALL) and B-cell Non-Hodgkin Lymphoma (B-NHL) | CD19 | Precursor Cell Lymphoblastic Leukemia-Lymphoma; Non-hodgkin's Lymphoma | Ⅰ; Ⅱ | Child, Adult | 21 | United States; France; Germany; Italy; Netherlands; Spain | Terminated | Absence of significant therapeutic benefit over existing therapies | DLT（up to 28 days after JCAR017 infusion）: 27.3%; ORR(Up to Day 56): 45.5%; DOR(0.05×10^6 CAR+ T Cells/kg,up to approximately 14 months): 13.7months; EFS(Up to approximately 15 months): 4.0months | SAE: 64.3%; Other (Not Including Serious) Adverse Events: 100.0% |
| 49 | NCT03744676 | A Safety Trial of Lisocabtagene Maraleucel (JCAR017) for Relapsed and Refractory (R/R) B-cell Non-Hodgkin Lymphoma (NHL) in the Outpatient Setting (TRANSCEND-OUTREACH-007) | CD19 | Non-hodgkin's Lymphoma; B-cell Lymphoma; Diffuse LBCL; Lymphoproliferative Disorders | Ⅱ | Adult, Older adult | 104 | United States | Completed | / | CRS Adverse Events Grade ≥ 3(up to approximately 90 days): 0.0%; NT Adverse Events Grade ≥ 3(up to approximately 90 days): 9.8%; Infection Adverse Events Grade ≥ 3(up to approximately 90 days): 11.0%; Prolonged Grade ≥ 3 Cytopenia at Day 29（At Day 29 after first treatment）: 32.9%; Response Rate（From first dose to up to approximately 41 months）: ORR: 80.5%，CRR: 53.7%，PFS: 5.83%，OS: 22.0% | SAE: 58.6%; Other (Not Including Serious) Adverse Events: 100.0% |
| 50 | NCT03761056 | Study to Evaluate the Efficacy and Safety of Axicabtagene Ciloleucel as First-Line Therapy in Participants With High-Risk Large B-Cell Lymphoma | CD19 | B-cell Lymphoma | Ⅱ | Adult, Older adult | 42 | United States; Australia; France | Completed | / | Response Rate Per the Lugano Classification as Determined by Study Investigators（Up to 4 years）: CR: 86.0%，ORR: 92.0%; CNS Disease（Up to 4 years）: 0.0% | SAE: 56.1%; Other (Not Including Serious) Adverse Events: 100.0% |
| 51 | NCT03873805 | PSCA-CAR T Cells in Treating Patients With PSCA+ Metastatic Castration Resistant Prostate Cancer | PSCA, CD19 | Castration-Resistant Prostate Carcinoma; Metastatic Prostate Carcinoma; Stage IV Prostate Cancer AJCC v8 | Ⅰ | Adult, Older adult | 14 | United States | Active not recruiting | / | DLT（Up to 28 days post treatment）: 14.3% | SAE: 57.1%; Other (Not Including Serious) Adverse Events: 100.0% |
| 52 | NCT03958656 | T-cells Expressing an Anti-SLAMF7 CAR for Treating Multiple Myeloma | SLAMF7 | MM; Plasma cell myeloma | Ⅰ | Adult, Older adult | 13 | United States | Completed | / | Response（At two and five weeks for stable disease and partial remission, respectively, and up to 5 months and 23 days for progressive disease）: PR（at 5 Weeks）: 11.1%，Stable Disease（at 2 Weeks）: 55.6%，Progressive Disease: 33.3% | SAE: 30.0%; Other (Not Including Serious) Adverse Events: 100.0% |
| 53 | NCT04002401 | Safety and Efficacy of Axicabtagene Ciloleucel in Combination With Rituximab in Participants With Refractory Large B-Cell Lymphoma | CD19 | Refractory LBCL | Ⅱ | Adult, Older adult | 27 | United States | Completed | / | CRR Per the International Working Group (IWG) Lugano Classification as Determined by Study Investigators（First infusion date up to maximum duration of 32.7 months）: 73.0%; PFS Per the IWG Lugano Classification as Determined by Study Investigators(up to approximately 32.7 months): 23.6 months; OS（up to approximately 32.7 months): NA | SAE: 53.9%; Other (Not Including Serious) Adverse Events: 100.0% |
| 54 | NCT04030195 | Dose-escalation Study of Safety of PBCAR20A in Subjects With r/r NHL or r/r CLL/SLL | CD20 | R/R Non-Hodgkin's Lymphoma; Relapse Chronic Lymphoid Leukemia; Small Lymphocytic Lymphoma | Ⅰ; Ⅱ | Adult, Older adult | 18 | United States | Completed | / | MTD（Day 1 to Day 28）: NA; Number of Participants With DLT（1 year）: 5.6%; Objective Response（1 year）: CR: 5.6%，PR: 27.8%，Non-responders: 66.7%; PFS（1 year）: 29.2 days | SAE: 23.5%; Other (Not Including Serious) Adverse Events: 100.0% |
| 55 | NCT04097301 | Study of CAR T-cell Therapy in Acute Myeloid Leukemia and Multiple Myeloma | CD44v6 | Acute Myeloid Leukemia; MM | Ⅰ; Ⅱ | Child, Adult, Older adult | 8 | Czechia; Italy | Terminated | Inability to close the study in a clinically relevant time frame | Phase I: Absence of RCR in Blood Specimens（3 Months Post-infusion）: 100.0%; Phase IIa: Hematological Disease Response to MLM-CAR44.1 T-cells in MM（3 months after T-cell infusion, assessed as day 0）: 0.0% | SAE: 100.0%; Other (Not Including Serious) Adverse Events: 100.0% |
| 56 | NCT04160195 | T Cells Expressing Fully-human Anti-CD19 and Anti-CD20 Chimeric Antigen Receptors for Treating B-cell Malignancies and Hodgkin Lymphoma | CD19, CD20 | B-cell Lymphoma; Non-hodgkin's Lymphoma; Chronic Lymphocytic Leukemia | Ⅰ | Adult, Older adult | 2 | United States | Terminated | The original principal investigator left the National Institutes of Health (NCI) and the decision was made to close the study to enrollment. | DLT(First protocol treatment through 28 days after the CAR T-cell infusion): 100.0%; CR(Approximately 1 year 5 months): 0.0%; PR(Approximately 1 year 5 months): 100.0% | SAE: 100.0%; Other (Not Including Serious) Adverse Events: 100.0% |
| 57 | NCT04225676 | Study of Efficacy and Safety of Reinfusion of Tisagenlecleucel in Pediatric and Young Adult Patients With Acute Lymphoblastic Leukemia (ALL) | CD19 | ALL | Ⅱ | Child, Adult | 5 | United States | Terminated | slow enrollment | Percentage of Patients Who Establish B Cell Aplasia Within 9 Months of Reinfusion: 40.0%; CR or CRi by Day24: 10.0%; OS(up to 9 months): 0.0% | SAE: 60.0%; Other (Not Including Serious) Adverse Events: 80.0% |
| 58 | NCT04314843 | Study of Lenzilumab and Axicabtagene Ciloleucel in Participants With Relapsed or Refractory Large B-Cell Lymphoma | CD19 | R/R LBCL | Ⅰ | Adult, Older adult | 6 | United States | Terminated | safety concerns | Phase 1: Percentage of Participants Experiencing AEs Defined as DLT Related to Sequenced Therapy With Lenzilumab and Axicabtagene(First infusion of lenzilumab and axicabtagene ciloleucel up to 28 days): 0.0% | SAE: 83.3%; Other (Not Including Serious) Adverse Events: 100.0% |
| 59 | NCT04436029 | Descartes-11 Consolidation Treatment in Patients With High-Risk Multiple Myeloma Who Have Residual Disease After Induction Therapy | BCMA | MM | Ⅱ | Adult, Older adult | 5 | United States | Completed | / | Stringent CR(1 year): 25.0% | SAE: 0.0%; Other (Not Including Serious) Adverse Events: 0.0% |
| 60 | NCT04816526 | Descartes-08 Consolidation Treatment in Patients With High-Risk Multiple Myeloma High-Risk Multiple Myeloma Who Have Residual Disease After Induction Therapy | BCMA | MM | Ⅱ | Adult, Older adult | 13 | United States | Terminated | Sponsor decision | Stringent CR: 53.8% | SAE: 7.7%; Other (Not Including Serious) Adverse Events: 84.6% |
| 61 | NCT05347485 | A Study of JNJ-68284528 Out-of-Specification (OOS) for Commercial Release in Participants With Multiple Myeloma | BCMA | MM | Ⅱ | Adult, Older adult | 86 | United States | Completed | / | ORR(up to 18.6 months): 57.3%; PR(up to 18.6 months): 9.8%; CRR(up to 18.6 months): 17.1%; PFS(up to 18.6 months): NA | SAE: 51.2%; Other (Not Including Serious) Adverse Events: 98.8% |
| 62 | NCT05588440 | A Clinical Study of ONCT-808 in Subjects With Relapsed or Refractory B-Cell Malignancies | ROR1 | R/R Aggressive B-Cell Malignancies | Ⅰ; Ⅱ | Adult, Older adult | 9 | United States | Terminated | Based on the available clinical data and capital requirements for continued development | DLT(Up to 28 days): 16.7%; Best Metabolic Response Rate Post-Baseline(up to 1 year): Complete Metabolic Response: 33.3%, Partial Metabolic Response: 16.7%, Not Done: 50.0% | SAE: 66.7%; Other (Not Including Serious) Adverse Events: 100.0% |

Here is the count of participants with serious and non-serious adverse events assessed by the Common Terminology Criteria in Adverse Events (CTCAE v3.0). A non-serious adverse event is any untoward medical occurrence. A serious adverse event is an adverse event or suspected adverse reaction that results in death, a life threatening adverse drug experience, hospitalization, disruption of the ability to conduct normal life functions, congenital anomaly/birth defect or important medical events that jeopardize the patient or subject and may require medical or surgical intervention to prevent one of the previous outcomes mentioned.

NA, not applicable;

ND, no data

CR, Complete Remission

PR, Partial Remission

PFS,Progression-free Survival

OR, Objective Response

DLT, Dose Limiting Toxicities

OS, Overall Survival

CRi, CR with incomplete blood count recovery

ORR, Overall Response Rate

MRD, Minimal Residual Disease

CRR, Complete Response Rate

EFS, Event-free Survival

RFS, Relapse Free Survival

DOR, Duration of Response

CRS, Cytokine Release Syndrome

NT, Neurotoxicity

MTD, Maximum Tolerated Dose

RCR, Replication Competent Retrovirus

AEs, Adverse Events
